# BAD overexpression inhibits cell growth and induces apoptosis via mitochondrial-dependent pathway in non-small cell lung cancer

**DOI:** 10.1186/1475-2867-13-53

**Published:** 2013-06-01

**Authors:** Li Jiang, Man Luo, Dan Liu, Bojiang Chen, Wen Zhang, Lin Mai, Jing Zeng, Na Huang, Yi Huang, Xianming Mo, Weimin Li

**Affiliations:** 1 Department of Respiratory Medicine, West China Hospital, Sichuan University, Chengdu 610041, P.R China; 2 Department of Respiratory Medicine, Nanchong Central Hospital, Nanchong 637000, P.R China; 3 Department of Respiratory Medicine, the First Affiliated Hospital of Chengdu Medical College, Chengdu 610072, P.R China; 4 Clinical Laboratory Department, Sichuan Academy of Medical Sciences and Sichuan Provincial People’s Hospital, Chengdu 610072, P.R China; 5 Laboratory Stem Cell Biology, State Key Laboratory of Biotherapy, West China Hospital, Sichuan University, Chengdu 610041, P.R China

**Keywords:** BAD protein, Non-small cell lung cancer, Apoptosis, Cell proliferation, Overexpression

## Abstract

**Background:**

The pro-apoptotic Bcl-2 protein BAD initiated apoptosis in human cells and has been identified as a prognostic marker in non-small cell lung cancer (NSCLC). In this study, we aimed to explore the functions of BAD in NSCLC.

**Methods:**

Overexpression of BAD was performed by transfecting different NSCLC cell lines with wild-type BAD. Cell proliferation, cell cycle, apoptosis, and invasion were characterized *in vitro*. Tumorigenicity was analyzed *in vivo*. Western blot was performed to determine the effects of BAD overexpression on the Bcl-2 family proteins and apoptosis-related proteins.

**Results:**

Overexpression of BAD significantly inhibited cell proliferation in H1299, H292, and SPC-A1 but not in SK-MES-1 and H460 cell lines *in vitro*. BAD overexpression also reduced the tumorigenicity of H1299/SPC-A1 cell *in vivo*. However, no appreciable effects on cell cycle distribution and invasion were observed in all these cell lines. BAD overexpression also induced apoptosis in all cell types, in which process expression of mitochondrial cytochrom c (cyto-c) and caspase 3 were increased, whereas Bcl-xl, Bcl-2, Bax and caspase 8 expressions did not changed. These findings indicated that a mitochondrial pathway, in which process cyto-c was released from mitochondrial to activate caspase 3, was involved in BAD overexpression-mediated apoptosis.

**Conclusions:**

Our data suggested that increased expression of BAD enhance apoptosis and has negative influence on cell proliferation and tumor growth in NSCLC. Bad is a new potential target for tumor interventions.

## Background

Lung cancer is the most frequent cancer worldwide with the highest cancer-related mortality [1]. The American Cancer Society estimates that in 2013, there would be 159,480 deaths from lung cancer and 228,190 new cases diagnosed. Of these, non-small cell lung cancer (NSCLC) account for ~ 85%. Currently therapeutic interventions have little influence on the prognosis of patients with NSCLC. The 5-year survival rates remain less 15% and recurrence rate still increased after surgery [2]. Mechanism that enable cancer cell to evade apoptosis may contribute to therapeutic resistance, which is a major challenge for prognostic improvement in NSCLC. Thus, anticancer agents triggered the cell undergoing apoptosis could improve response to treatment and clinical outcome. We recently reported that the BAD (Bcl-Xl/Bcl-2-associated death promoter homologue), as a pro-apoptotic Bcl-2 protein, shows decreased expression level, and plays an important role for predicting therapeutic response and survival in NSCLC [3].

BAD, as one of the “death-promoting” members of the Bcl-2 family [4], is activated through dephosphorylation responsible for apoptotic event, in contrast, inactivation promote cell survival through phosphorylation on several serine residues by upstream kinase, such as Akt, Raf, Pim-2, and PKA [3,5]. It is largely assumed that BAD interact with pro-survival Bcl-2 family proteins, including Bcl-2 and Bcl-xl, to ablate their pro-survival function. This ablation allows activation of downstream, such as Bax and Bak,to induce cell to apoptosis [6,7]. BAD protein has been reported to contribute to tumorigenesis and chemotherapy resistant. Ranger et al. found that BAD-deficient mice develop diffuse large B cell lymphoma [8]. BAD^−/−^ mammary cancer cells are resistant to gefitinib-therapy in the study by Gilmore [9]. Moreover, BAD have been shown to be prognostic biomarkers for colon cancer, ovarian cancer, and breast cancer patients [10,11]. Our previous studies also provided clinical evidence that loss of BAD is an independent and powerful predictor of adverse prognosis in NSCLC [3]. Therefore, in addition to regulating apoptosis, BAD might be involved in various cellular functions, such as proliferation and tumor growth in NSCLC.

In this study, our data provided experimental evidence that BAD could play functions as a tumor suppressor in NSCLC. Increased BAD expression has effects on proliferation of NSCLC cell lines and tumor growth *in vivo*. Meanwhile, BAD overexpression induced apoptosis in all cell types, in which process cytochrom c (cyto-c) and caspase 3 releases was involved.

## Methods

### Cell culture and animal models

The following cell lines were obtained from the Type Culture Collection of the Chinese Academy of Sciences and cultured according to recommendations: NCI-H1299 (metastatic NSCLC, H1299), NCI-H292 (mucoepidermoid adenocarcinoma cell, H292), NCI-H460 (large cell carcinoma cells, H460), SPC-A1 (pulmonary adenocarcninoma cells), and SK-MES-1 (squamous cell carcinoma cells, SK-MES). H1299/H292/H460/SPC-A1/SK-MES-BAD cells (NSCLC cell-BAD) were generated by transfecting those cell lines with wild-type BAD (Pdonr223/BAD, Neuron Biotech, Shanghai, China) as previously described [12]. H1299/H292/H460/SPC-A1/SK-MES-NC cells (NSCLC cell-NC) refer to the cell lines transfected with empty vector (pLOV. UBC. EGFP, Neuron Biotech, Shanghai, China). All cultured cells were maintained in a humidified 5% CO2 atmosphere at 37°C. Nude, 5-8-week-old athymic nude mice (BALB/c-nu/nu nude mice), half of which were female and half male were obtained from the Laboratory Animal Centre of Sichuan University. All mice were housed in laminar flow cabinets under specific, pathogen-free conditions with food and water provided *ad libitum*. All animal procedures listed in this article were performed in accordance with the Helsinki Convention for the use and care of animals, and approved by the Institutional Animal Care and Treatment Committee of Sichuan University.

### Tissue protein extraction and western blot assay

Total protein was extracted from cultured cells and xenograft tumours using the whole protein extraction kit (KeyGEN, Nanjing, China), Protein concentration were measured using BCA Protein Assay Reagent (Thermo scientific, Rockford, USA). Equivalent amounts of protein from different samples were subjected to sodium dodecyl sulphate–polyacrylamide gel electrophoresis (SDS-PAGE) using polyvinylidene fluoride (PVDF) membranes (Millipore, Billeraica, USA) to electro blot. The membranes were incubated overnight at 4°C with anti-BAD monoclonal antibody (#9292, 1;1000, Cell Signaling Technology, Beverly, USA), Bcl-xl (#24247, 1:1000, Signalway Antibody, Pearland, USA), Bcl-2 (#24246, 1:1000, Signalway Antibody), Bax (#24250, 1:1000, Signalway Antibody), Caspase 3 (#21420, 1:1000, Signalway Antibody), Caspase 8 (#21421, 1:1000, Signalway Antibody), cytochrome C (#21680, 1:1000, Signalway Antibody) and β-actin (#4970, 1:5000, Cell Signaling Technology). Target proteins were detected using the ChemiDoc XRS system (Bio-rad, Philadelphia, USA) by exposure to chemiluminescent HRP substrate (Millipore, Billerica, USA) and analyzed via Quantity One 1-D Analysis software (Bio-rad) [3,13].

### Cell proliferation assay

The effects of BAD on the proliferation of NSCLC cell lines were determined using Cell Counting kit-8 (CCK-8; Dojindo, Kumamoto, Japan) according to the manufacturer’s protocol. Briefly, cells were seeded in 96-well plates at a density of 2 × 10^3^ per well. The plates were incubated at 37°C for 1, 2, 3, 4, 5 and 6 days. Then the optical density was measured at 450 nm to determine cell proliferation index. Results are presented as the mean ± SD based on at least three independent experiments.

### Cell cycle analysis

Approximately 1 × 10^6^ cells were harvested and washed 3 times with PBS and switch to media containing 0.05% fetal bovine serum (FBS). Then the cells were re-suspended in DNA staining solution [(PI, 50 μg/ml), RNase A (100 μg/ml)), and 0.1% (vol/vol) Triton X-100 in PBS) and incubated at room temperature for 30 min. The DNA content was determined by FACS Calibar Flow Cytometer (Becton-Dickinson, Franklin Lakes, USA). Data were analyzed using CellQuest and Modfit software.

### Invasion assay

The invasion of NSCLC cell lines were measured using the BD BioCoat Tumor Invasion Assay System (BD Bioscience) according to the manufacture’s protocol. Cell suspensions containing 1 × 10^6^ cells/ml were seeded onto the upper chamber with serum free media. DMEM containing 10% FBS, as a chemoattractant, was then added to the lower chamber. After 48 h of incubation, the invasive cells on the lower surface of the membrane were stained by dipping the inserts into staining solution for 20 min. The cells were measured by photographing the membrane using a microscope at five random views.

### Apoptosis assessment

Approximately 5 x 10^5^ cells were harvested, washed in PBS and incubated with 5 μl Annexin V-APC and 7AAD (KeyGen, Nanjing, China) at room temperature for 5 min. Fluorescence was measured using a FACS Calibar Flow Cytometer. The Annexin V-positive and 7AAD-negative cells were regarded as apoptotic.

The TUNEL (terminal deoxynucleotidyl transferase-mediated dUTP nick end labeling) assay was performed to detect early stage of DNA fragmentation in apoptotic cells by using a commercial kit (*In situ* Cell Death Detection kit, TMR red, Roche Diagnostics Limited, Shanghai, China). Paraffin-embedded xenograft tumor sections were permeabilized in 0.1% Triton X-100, and then incubated with TUNEL reaction mixture containing TdT and TMR-dUTP. During this incubation step, TdT catalyzes the attachment of TMR-dUTP to free 3’OH ends in the DNA. Nuclei were counterstained with DAPI. Tissue sections were analyzed for apoptotic cells with localized TMR red under a fluorescence microscope. The apoptotic cell rate was determined according to the formula: (number of apoptotic cell with red staining/total number tumor cells with DAPI staining) × 100%.

### Immunohistochemistry

IHC assay was performed on histological sections of formalin-fixed tumor xenografts as previously described [3,14]. The primary antibody is proliferative marker ki-67 (1:100, Dako). The positive cell rate was measured using a microscope at five random views.

### Assessment of tumorigenicity *in vivo*

All kinds of treated H1299 and SPC-A1 cells were harvested (1 × 10^6^cells in 150 μl) and injected subcutaneously in athymic nude mice. Mice were divided into three groups, including H1299/SPC-A1, H1299/SPC-A1-BAD overexpression (H1299/SPC-A1-BAD), and BAD-negative control (H1299/SPC-A1-NC). Four weeks later, mice were sacrificed, and tumors were dissected and weighed, then fixed using formalin or stored at −80°C until further use. Tumor volume (mm^3^) = 0.52 × length (mm) × width (mm) × width (mm). Each group contains 6 mice.

### Statistical analysis

Pearson chi-square tests were used to assess the difference in protein expression status among cell lines and tissues. Analysis of variance was performed to determine the statistical significance of differences among the experimental groups. All values were expressed as means ± SD, and Levels of statistical significance were set at P < 0.05 (two-sided). All data were analyzed using SPSS 13.0 for Windows (SPSS Inc., Chicago, Ill, USA).

## Results

### Overexpression of BAD in NSCLC cell lines inhibits cell proliferation *in vitro* and tumor growth *in vivo*

**Figure 1:**
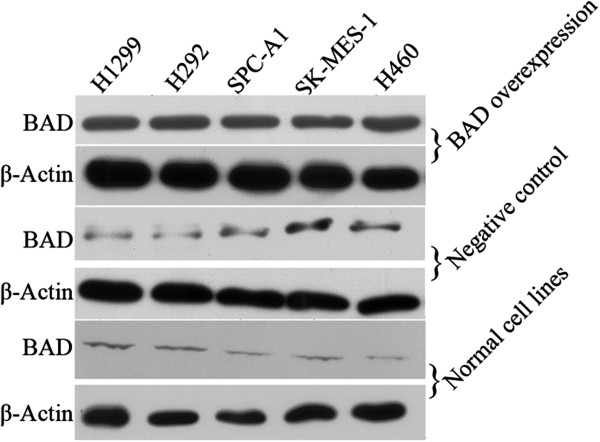
**BAD overexpression in NSCLC cell lines and xenograft tumor tissues.** Total proteins were extracted from different cell lines. The protein levels of BAD were analyzed by Western blot. β-Action was used to normalize relative expressions among groups.

To determine whether high levels of BAD expression contributed to the NSCLC cell proliferation, invasion and apoptosis, we employed a BAD-expressing vector Pdonr223/BAD-pLOV. UBC. EGFP to transfect BAD into NSCLC cell lines, including H1299, H292, A549, H460 and SK-MES. As shown in the Figure 1, expression level of BAD drastically increased in transfected cell groups compared with the non-transfected and empty vector-transfeced cell group (all p < 0.05). In the NC group, BAD expression was not affected by unrelated vector.

**Figure 2:**
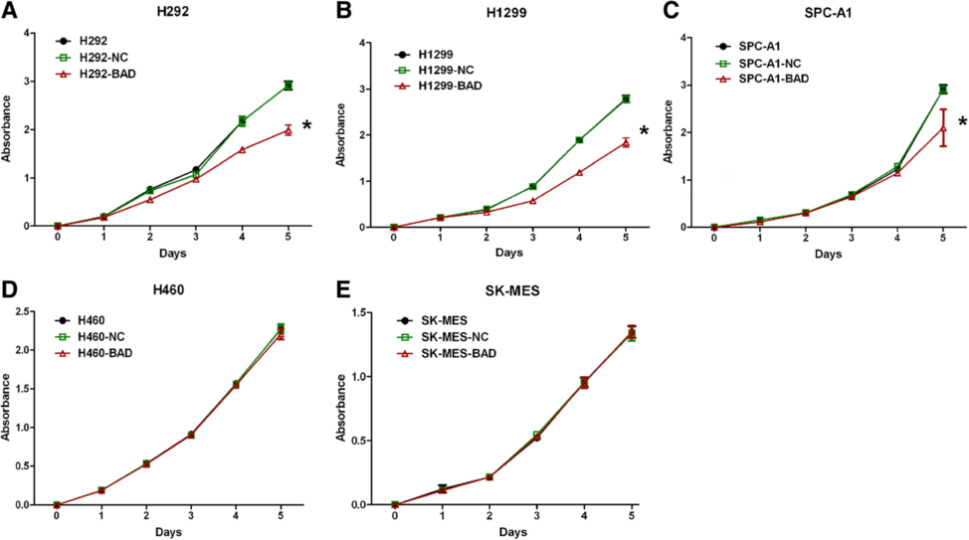
**Effects of BAD overexpression on NSCLC cells proliferation.** Cell proliferation rates were examined using Cell counting kit-8 at 1, 2, 3, 4, 5 and 6 days. The optical densities were measured at 450 nm to compare cell viability among groups. *: p < 0.05.

**Figure 3:**
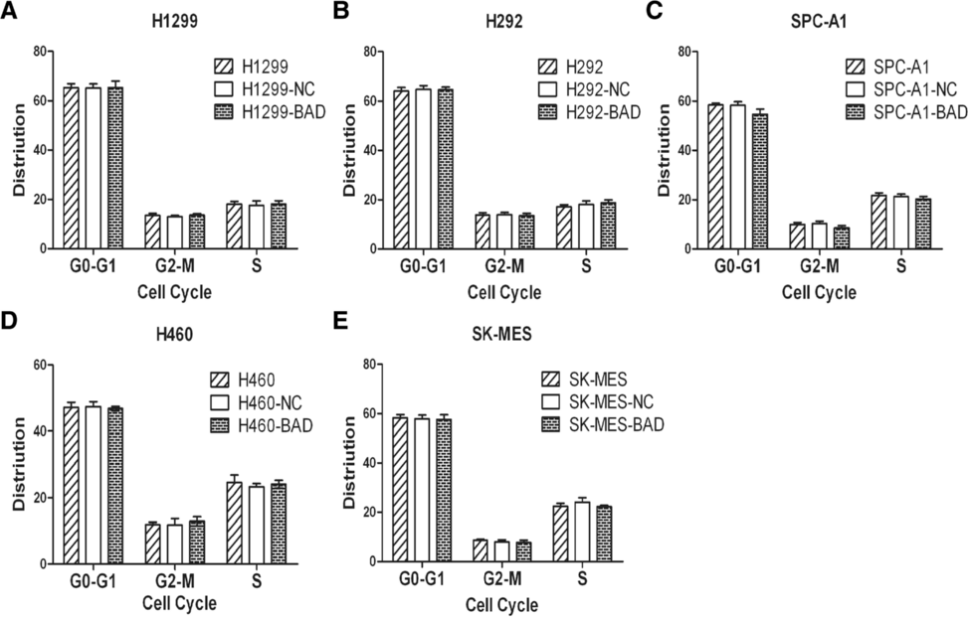
**Effects of BAD overexpression on NSCLC cells cycle.** Approximately 5 × 10^5^ to 1 × 10^6^ cells were harvested and stained using propidium iodide. DNA content was determined by flow cytometry. Aggregated cells were gated out and cell cycle profiles were analyzed to quantitate cell cycle distribution. No significant effects on the cell cycle distribution were observed in all cell lines (all p > 0.05).

Cell proliferation was examined using Cell counting kit-8 at the time points of 1, 2, 3, 4, 5, and 6 days. The cell proliferation rates of the BAD overexpression cell group were significantly decreased compared with NC and normal cell lines in H292, H1299, and SPC-A1, respectively (Figure 2A-C, all p < 0.01), while showed a trend toward lower proliferation rate in H460 cell line, although the difference did not reach statistical significance (Figure 2D, p = 0.077). However, no differences were observed when SK-MES-1 cell line was transfected (Figure 2E, p > 0.05)), suggesting that BAD overexpression inhibited cell proliferation except for squamous cancer cell line. In cell cycle analysis, our results demonstrated that no significant effect on the cell cycle distribution was observed in all cell lines (Figure 3, all p > 0.05).

**Figure 4:**
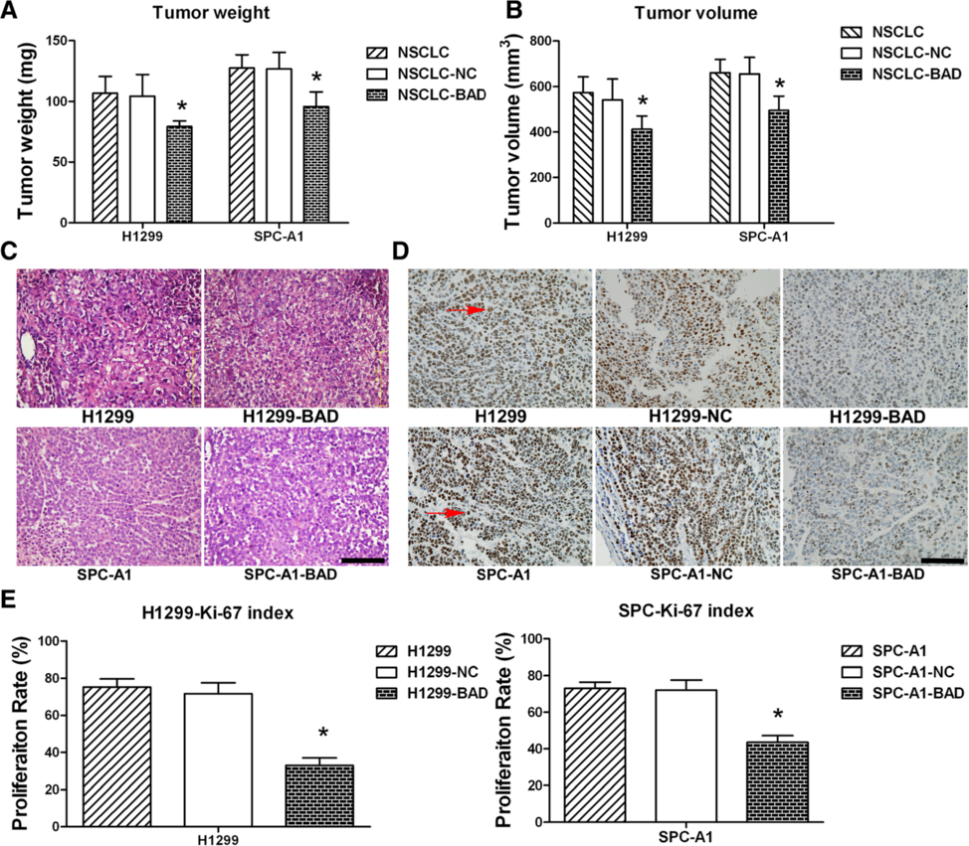
**BAD overexpression inhibits H1299/SPC-A1 cell tumorigenic activity.** Cells (1 × 10^6^ cells in 150 μl) were injected subcutaneously into the backs of BALB/c-nu/nu nude mice. After 4 weeks, tumour weights (**A**) and volumes (**B**) were significantly decreased in BAD overexpression groups compared with H1299/SPC-A1-NC and H1299/SPC-A1 groups. The xenograft tumors were processed for H&E staining (**C**). Representative examples of proliferative marker ki-67 staining in xenograft tumor tissues are shown (**D**). And data were represented as the mean Ki-67 index ± SD (**E**). Original magnification, × 200. Arrows indicate positive staining of nuclei. Bar: 50 μm. Data are expressed as the mean ± SD. *: p < 0.05.

To better understand the effects of BAD overexpression on NSCLC, xenograft models were established. After subcutaneous injection of H1299/SPC-A1, H1299/SPC-A1-BAD and H1299/SPC-A1-NC cells to the BALB/c-nunu nude mice, tumor volumes were measured every 3 days. Cells in H1299/SPC-A1 and NC group can rapidly form tumors. Nevertheless, BAD overexpression in H1299/SPC-A1 cells reduced tumor formation compared with H1299/SPC-A1 and H1299/SPC-A1-NC control (Figure 4A-B, all p < 0.01). H&E staining showed that xenograft tumor tissues retained major features of the original cancer (Figure 4B). In accordance with the slower growth of H1299/SPC-A1-BAD xenograft, immunohistochemical analysis showed less cells that stained positive for the proliferative marker Ki-67 than those of the controls (Figure 4C-D, all p < 0.01).

### Overexpression of BAD has no effect on cell invasion ability

Cell invasion assays were performed to determine the impact of BAD overexpression in NSCLC. We counted invasive cells that transferred to the lower surface of the membrane at five randomly located areas. In all cell types, BAD overexpression had no influence on cell invasion in NSCLC cell types (data not shown, all > 0.05).

### Overexpression of BAD induced cell apoptosis in NSCLC cell and xenograft tumors

**Figure 5:**
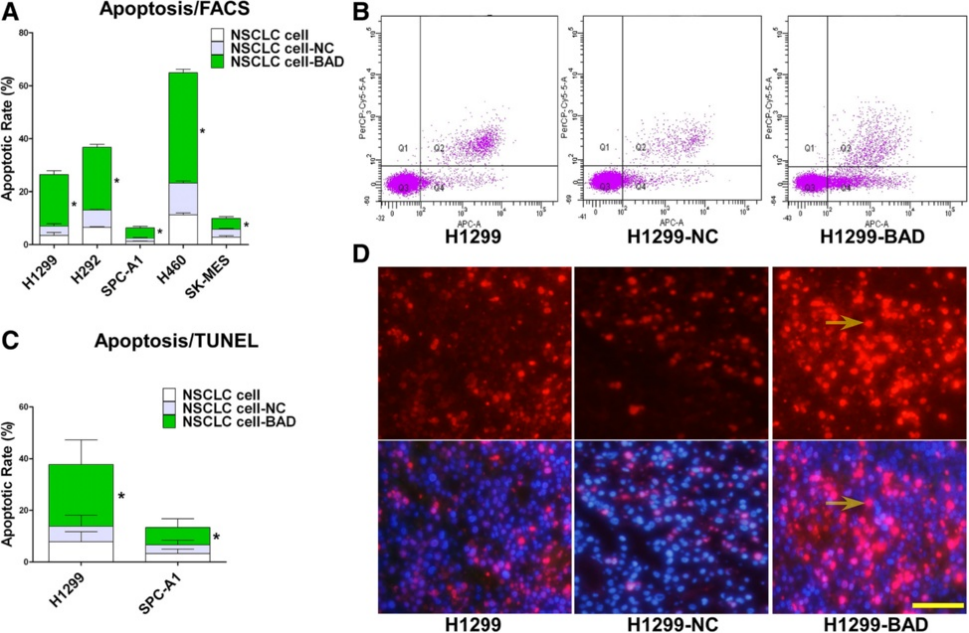
**Effects of BAD overexpression on NSCLC cell apoptosis.** Cells were harvested and stained with V-APC/7AAD. Apoptotic rates were caucluated by flow cytometry (**A**). The Annexin V-positive and 7AAD-negative cells (Q4) were regarded as apoptotic (**B**). For the xenograft mice, paraffin-embedded sections of H1299 and SPC-A1 tumor were subjected to TUNEL assay. The amount of TUNEL fluorescence in five random view was quantified (**C**, all p < 0.05). Representative photomicrographs of TUNEL staining were shown (**D**). Apoptotic neuclei showed red staining (top), and nuclei stained with DAPI in blue (bottom). The merged images of nuclei in pink (arrows) indicate apoptotic cells. Original magnification, × 200, Bar: 50 μm.

Apoptotic rates were determined by flow cytometry and TUNEL assays. Our results showed that overexpression of BAD markedly promoted cell apoptosis. In H1299, H292, H460, SPC-A1, and SK-MES-1 cell lines overexpressing BAD, the average apoptotic rates were 19.42%, 23.70%, 41.72%, 3.92%, and 3.12%, respectively, which were higher than those of the control groups (Figure 5A-B, all < 0.05), especially in H460-BAD group (p = 0.000).

For the xenograft mice, paraffin-embedded sections of H1299 and SPC-A1 tumor were also subjected to TUNEL assay. As shown in Figure 5D, apoptotic cells showed red staining using an inverted fluorescence microscope. The results indicated that the apoptotic cell rates were higher in H1299-BAD and SPC-A1-BAD groups than the controls by >2-fold (Figure 5C, p < 0.05). All data suggested BAD overexpression significantly enhanced tumor cell apoptosis.

### Overexpression of BAD enhanced the cyto-c and caspase 3 expressions

**Figure 6:**
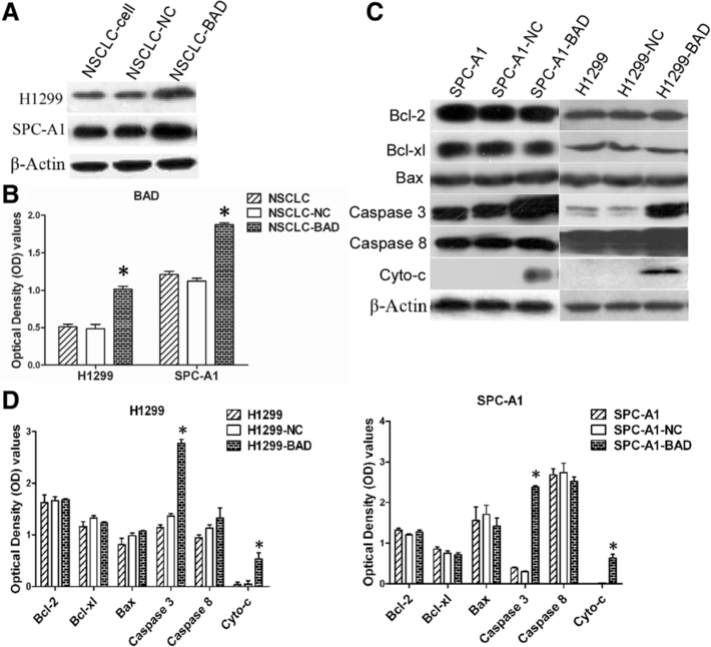
**The expressions of Bcl-2 family proteins and apoptotic related proteins after BAD overexpression.** Western blot analysis confirmed that BAD were overexpressed in xenograft model (**A**-**B**). The expression levels of Bcl-2, Bcl-xl, Bax, caspase 3, caspase 8 and cyto-c after BAD overexpression were analyzed (**C**-**D**, from top to bottom). β-Actin was used to normalize relative expressions among groups.

In xenograft tumor tissues, the expression levels of BAD were increased in overexpression groups compared with H1299/SPC-A1 and H1299/SPC-A1-NC cell groups (Figure 6A-B).

As BAD is one of core members of the Bcl-2 family, we further analyzed the effect of BAD on expression of other Bcl-2 family members, including Bcl-xl, Bcl-2, Bax, however, all protein above remained unchanged. Meanwhile, expression levels of apoptosis related proteins, such as cyto-c, caspase-3, and caspase-8, were also determined by western blot. Among the proteins aforementioned, cyto-c and caspase 3 expressions were significantly higher in the BAD overexpression group than the controls (all p < 0.05); whereas the other proteins showed similar expression levels in different groups (Figure 6C-D, all p > 0.05).

## Discussion

BAD, an important regulator of the cell death machinery, has been reported to contribute to tumorigenesis in several cancers [10,15,16]. Loss-of-function models of BAD protein are associated with increased incidence of tumors, and over time, BAD-deficient mice show an increased death rate from tumors [17]. In light of these findings, tumor therapy with BAD protein up-regulation may be warranted. In the present study, we demonstrated that up-regulation of BAD significantly reduced cell proliferation in H1299, H292 and SPC-A1 cell lines *in vitro* and H1299/SPC-A1 cell tumor growth *in vivo*, but has no appreciable effects on cell cycle distribution and invasion (data not shown, all p > 0.05) in all cell lines. BAD overexpression also induced apoptosis in all cell types, of which process cyto-c and caspase 3 was involved.

BAD protein, as one of cell death initiators, constitutes a critical control point in apoptosis following cellular damage. Loss of BAD expression alone may promote tumorigenesis due to lack of apoptosis and accumulation of cells with tumorigenic potential. In our study, overexpression of BAD was able to greatly increase the apoptotic rates of NSCLC cell in cultured cells and xenograft tissues, confirming its proapoptotic nature. These are keeping with the report by Mork et al. [18] that BAD act as a key regulator of T cell apoptosis and BAD transgenic mice have depleted numbers of T cells by enhancing sensitive to apoptotic stimuli. Agshin et al. [19] also found that prostatic carcinoma cell line LNCaP, which was resistant to tumor necrosis factor-related apoptosis-inducing ligand (TRAIL)-induced apoptosis, became sensitive to TRAIL and promote apoptotic rate after overexpression of full-length, wild-type BAD. [18]. However, the apoptotic effect of BAD protein overexpression is still controversial. Several studies have shown that overexpression of BAD alone in a cell line has no effect on apoptosis, whereas coexpressing of BAD and Bcl-xl significantly influence cell apoptosis, suggesting that the action of BAD is dependent on heterodimerization with Bcl-xl [20,21]. Thus, we proposed that the effect of BAD on apoptosis is a result of regulation and interaction with other Bcl-2 family members, including Bcl-xl, Bcl-2, and Bax. Unexpectedly, all these protein expression levels were unchanged. These indicated that BAD could function as a powerful regulator of apoptosis in NSCLC cell independent of Bcl-xl/Bcl-2/Bax interactions or expressions.

Two major apoptotic pathways, the death receptor (extrinsic pathway) and the mitochondrial pathway (intrinsic pathway), have been well characterized in mammalian cells. Over the cause of these pathways, activation of the death receptor first triggers caspase-8 activation, whereas the release of mitochondrial cyto-c activates caspases-9 as an initial caspase 9, all of which subsequently induced the activation of effectors caspases, such as caspase 3 [22,23]. Cyto-c is an important mitochondrial protein that induces apoptosis when accumulated in the cytosol in response to diverse stress stimuli [24]. In our study, the results revealed that cyto-c and caspase 3 were increased by overexpressing BAD, whereas caspase 8 did not change. These indicated that BAD overexpression-induced apoptosis is associated with cyto-c releasing from mitochondrial, independent of caspase 8 activation. These are in accordance with the studies by Cheng [25] and Wei [26] that BH3 domain-only molecules, including BAD, were required for the disruption of mitochondrial and intrinsic death of cancer cells [27]. Thus, we suggested that BAD overexpression in NSCLC led cancer cells to undergo apoptosis through a mitochondrial pathway.

The pro-apoptotic Bcl-2 family proteins Bax and Bak have been shown to be required for the disruption of mitochondrial and intrinsic death of cancer cells, where as the antiapoptotic Bcl-2 family proteins (Bcl-2 and Bcl-xl) can prevent cell death by interfering with the activation of Bax and Bak [22]. In the present study, we found that the expression levels of Bax, Bcl-2 and Bcl-xl were not changed after BAD overexpression. These suggested that the Bad overexpression-induced mitochondrial pathway was independent of Bax, Bcl-2 and Bcl-xl expression levels.

Recent investigations suggest that functions of the proapoptotic protein BAD are not limited regulating apoptosis [21]. We also found that high level of BAD protein inhibited cell proliferation in H1299, H292, SPC-A1 cell line, and reduced H1299 tumor growth rate in immunocompetent mice. Further immunohistochemical analysis showed that xenograft tumor with BAD overexpression had a decreased number of cells that stained positive for the proliferative marker Ki-67. For H460, a large cell lung cancer cell line, the result also showed a trend toward lower proliferation rate, although the difference did not reach statistical significance. All these indicated that BAD play a negative role in specific cell types, especially in adenocarcinoma cells. This is keeping with our previous report that overexpression of BAD suppressed cell proliferation in another lung adenocarcinoma cell line A549 [12]. In breast cancer cell MCF7, cell growth was also inhibited by BAD overexpression [21]. Contradictive reports appeared that increased BAD expression stimulates proliferation of prostate cancer cells [28]. Knockdown of BAD also led to marked inhibition of proliferation in A375 and SK-MEL-28 malignant melanoma cells, and this growth inhibition could be abrogated by overexpression of wild type BAD [29]. Additionally, in our study, no differences of proliferation were observed in SK-MES-1 squamous cell lung cancer cell. These different results indicating that the effect of BAD on cell proliferation may be cell-type-specific.

To further characterize the mechanism underling growth inhibition, we performed cell cycle analysis. Publications from the Vogt and Yang laboratories have suggested that BAD protein can be involved in promoting cell cycle progression in fibroblast [28,30,31]. On the contrary, our results showed that overexpression of BAD did not influence cell cycle distribution in all NSCLC cells. These suggested that, in NSCLC, BAD inhibited cell proliferation *in vitro* and tumor growth *in vivo* through direct induction of apoptosis without affecting cell cycle progression.

In cell invasion analysis, our data demonstrated that BAD overexpression had no influence on cell invasion in NSCLC cell types. In contrast, a previous AACR symposium poster [32] reported that BAD inhibited cancer cell invasion in breast cancer. From now on, there are very limited reports of the effects of BAD on cell invasion. These inconsistencies remained to be confirmed in expanded and intensive studies.

## Conclusions

In conclusion, this study extended our previous findings that BAD expression level was an independent poor prognostic marker in NSCLC patients. BAD overexpression alone induces cell apoptosis, and depressed cell proliferation and cell growth depends on cell types, especially in adenocarcinoma. In the further investigation, BAD may function as tumor suppressor regulating cell growth and apoptosis in the development of NSCLC, and is a potential target for tumor intervention.

## Abbreviations

NSCLC: Non-small cell lung cancer; cyto-c: Cytochrom c.

## Competing interests

The authors declare that they have no competing interests.

## Authors’ contributions

JL and Man LM carried out most of the experimental studies. LD participated in designing the research, performed statistical analyses and drafted the manuscript. CBJ, ZW and ML participated in the animal and cell experiments. ZJ and HN participated in western blot assays. HY performed statistical analyses and drafted the manuscript. MXM revised the manuscript. LWM designed the research, supervised the experiments and completed the manuscript. All authors read and approved the final manuscript.
